# Disclosure and Exposure of Alcohol on Social Media and Later Alcohol Use: A Large-Scale Longitudinal Study

**DOI:** 10.3389/fpsyg.2017.01934

**Published:** 2017-11-01

**Authors:** Eilin K. Erevik, Torbjørn Torsheim, Cecilie S. Andreassen, Øystein Vedaa, Ståle Pallesen

**Affiliations:** ^1^Department of Psychosocial Science, University of Bergen, Bergen, Norway; ^2^Department of Clinical Psychology, University of Bergen, Bergen, Norway; ^3^Department of Health Promotion, Norwegian Institute of Public Health, Bergen, Norway

**Keywords:** social networking sites, social media, alcohol, exposure, disclosure, students

## Abstract

This article aims to investigate whether alcohol-related disclosure and exposure on social media can predict later alcohol use, and to identify covariates in these relationships. Data were collected by online surveys (two waves) among students in Bergen, Norway. The first survey was administered in fall 2015. The follow-up took place during fall 2016. A total of 5,217 students participated in both waves. The surveys included questions about demographics, personality, alcohol use, alcohol-related cognitions (e.g., attitudes and norms), social media use, and disclosure and exposure of alcohol on social media. Bivariate comparisons were conducted to assess differences in alcohol use between the frequent (i.e., monthly or more often) disclosure and exposure groups and low-frequent disclosure and exposure groups. Crude and adjusted linear regressions were employed to investigate if disclosure and exposure of alcohol could predict later alcohol use, when controlling for a range of covariates. Compared to the low-frequent disclosure and exposure groups, participants which frequently disclosed or were frequently exposed to alcohol-related content had higher alcohol use at baseline and 1 year later (*p* < 0.001), when no covariates were controlled for. Frequent disclosure of content reflecting positive aspects of alcohol predicted stable or slightly increased alcohol use at Time 2 (*p* < 0.01), even when all covariates (i.e., demographics, personality, alcohol use, alcohol-related cognitions, and social media use) were controlled for. In conclusion, frequent disclosure and/or exposure to alcohol-related content predicted alcohol use over time. Alcohol disclosure/exposure on social media could for the most part not predict later alcohol use when baseline alcohol use was controlled for. High alcohol use and alcohol disclosure/exposure on social media appear to be strongly intertwined, which hampers identification of directionality between alcohol use and disclosure/exposure. Disclosing content reflecting positive aspects of alcohol was the only independent variable that could predict further alcohol use when other factors, like baseline alcohol use, were held constant. This finding suggests that disclosure of alcohol content reflecting positive aspects of alcohol might have a self-enhancing effect on the sharers' further alcohol consumption, or that disclosing such content could indicate lenient alcohol-related cognitions not detected by the current measurements.

## Introduction

Alcohol use can cause much harm to the individual user as well as to society (Hingson et al., 2002; Rehm et al., 2003, 2009). Hence, identifying determinants of alcohol use is of public interest. Previous studies suggest that disclosure of alcohol-related content on social media indicates concurrent alcohol use (Westgate et al., 2014; Geusens and Beullens, 2016), while exposure have in some studies been found to predict later alcohol use (Huang et al., 2014b; Boyle et al., 2016). Social media could thus be an arena for detecting and preventing problematic alcohol use (Moreno et al., 2012; Moreno and Whitehill, 2014; Westgate et al., 2014). The association between disclosure and exposure and alcohol use may depend on type of content shared or seen (van Hoof et al., 2014; Westgate and Holliday, 2016; Groth et al., 2017), but the relationship between different types of alcohol-related content and alcohol use has not been investigated. The causal mechanisms underlying the relationship between alcohol-related disclosure and exposure and alcohol use are not fully understood (D'Angelo et al., 2014; Boyle et al., 2016) although it has been suggested that common factors (e.g., personality traits) may be at play. It has further been suggested that disclosure and exposure of alcohol-related content may be a mere reflection of alcohol use or that disclosure and exposure may instigate alcohol use (Moreno et al., 2012; Westgate and Holliday, 2016; Groth et al., 2017).

Certain characteristics seem to increase the likelihood of both disclosure and exposure of alcohol-related content on social media and high alcohol consumption (Westgate and Holliday, 2016). Status as single and extroversion are both positively associated with disclosure of alcohol-related content (Erevik et al., 2017) and alcohol consumption (Merenakk et al., 2003; Andersson et al., 2007; Raynor and Levine, 2009). Certain aspects of social media use, like the number of online-friends, seem also to be positively associated with disclosure and exposure and alcohol use (Egan and Moreno, 2011; Ridout et al., 2012; Beullens and Schepers, 2013; Moreno et al., 2014; Westgate et al., 2014; Westgate and Holliday, 2016). Associations between different aspects of social media use and alcohol use are, however, not consistently found (Hoffman et al., 2014; Huang et al., 2014b). Although studies on the relationship between alcohol disclosure and exposure on social media and alcohol use have controlled for factors such as gender, age, and number of online-friends (Glassman, 2012; Ridout et al., 2012), no previous study, thus far, has controlled for the wider range of demographic, personality, and social media factors that may influence this relationship.

Disclosure of alcohol-related content may be a direct reflection of the sharer's alcohol use (D'Angelo et al., 2014; Westgate et al., 2014; Geusens and Beullens, 2016). Disclosures could also be a reflection of alcohol-related social cognitions (i.e., perceived norms and attitudes), which are known to predict alcohol use (D'Angelo et al., 2014; Westgate et al., 2014). Moreover, exposure to alcohol-related content could be associated with alcohol-related social cognitions as well (Miller et al., 2014). Individuals with high alcohol intake and positive attitudes toward alcohol may be more attentive to alcohol-related content on social media, and hence more frequently exposed to such content as studies have shown stronger attention bias toward alcohol among heavy social drinkers than light social drinkers (Field et al., 2004). Frequent exposure to alcohol-related content may also indicate high alcohol intake in the individual's social network (Huang et al., 2014a), and peers' alcohol use is commonly known as a strong predictor of own alcohol use (Scholte et al., 2008). According to this line of reasoning, some studies have indicated that controlling for alcohol-related cognitions (i.e., normative apprehensions and attitudes) and/or alcohol use could weaken or eliminate the association between disclosure and exposure to alcohol-related content and future alcohol use (Huang et al., 2014a,b; Boyle et al., 2016).

Disclosure and exposure of alcohol-related content on social media have also been suggested to cause an increase in alcohol use (Huang et al., 2014b; Boyle et al., 2016; Groth et al., 2017). Disclosing content on social media could lead to a stronger commitment to continue to act in accordance with the attitudes and behaviors that were displayed in order to maintain a coherent self-image (Bem, 1972; D'Angelo et al., 2014). Receiving positive feedback (e.g., “likes”) on alcohol-related posts have been suggested to further enhance drinking through positive reinforcement mechanisms (Skinner, 1953; D'Angelo et al., 2014; Groth et al., 2017). Exposure to alcohol-related content has also been suggested to have direct causal effects on alcohol use (Litt and Stock, 2011; Fournier et al., 2013; Westgate and Holliday, 2016) through mechanisms such as mere exposure effects (Osberg et al., 2012), and indirectly through altering attitudes and perceived norms for alcohol use (Litt and Stock, 2011; Fournier et al., 2013; Boyle et al., 2016). The latter claim is supported by experimental studies showing that exposure to alcohol-related content on social media strengthened the receivers' perception of drinking norms among peers (Litt and Stock, 2011; Fournier et al., 2013).

Previous studies have linked disclosure and exposure to alcohol use, but few of these studies have been based on longitudinal or experimental designs (Boyle et al., 2016). The directionality between disclosure and exposure and alcohol use is therefore unclear. The current study sought to examine the longitudinal relationship between disclosure and exposure of different types of alcohol-related content on social media and later alcohol use, and to identify covariates that may explain these relationships.

## Materials and methods

### Procedures and sample

All students registered at the four largest institutions of higher education in Bergen municipality, Norway, were during fall 2015 invited (via e-mails) to participate in an online survey. A total of 11,236 (39.4%) agreed to participate. Participants from the first wave were invited to participate in a follow-up online survey during fall 2016. A total of 5,217 (51.5%) agreed to participate in the follow-up survey. The majority of the former participants were contacted by their student e-mails, while some were contacted by their private e-mails. Approximately 40% of the students ended their education between the first (Time 1, T1) and the second (Time 2, T2) wave of the survey of some reason (e.g., completion of academic degree), according to the institutions from which the sample is recruited. Based on the assumption that 25% of the former participants which were contacted by their student e-mail did not receive the invitation to participate in the follow-up survey, we estimated that about 61.2% of the ones who received an invitation agreed to participation. A rate of ~40% ending their education yearly may be a somewhat high rate in an international perspective. This might be related to the wide availability of higher education in Norway (i.e., due to loans/grants and the absence of tuitions) which may cause Norwegian students more likely to quit their education before completing a degree, as quitting will be less associated with financial loss compared to the cause in many other countries. The study protocol was approved by the Regional Committee for Medical and Health Related Ethics, Western Norway (no. 2015/1154), and the Norwegian Data Protection Authority (no. 49365). The study was conducted according to the principles expressed in the Declaration of Helsinki. In the beginning of each survey the participants were met with an informed content page, were they could choose whether or not they agreed to participation.

### Measurement

#### Demographics

Demographic variables were measured by questions about birth year, sex, place of birth, and religious identification at the first wave (2015). The participants were asked about relationship status, and parental status in both waves (Nedregård and Olsen, 2014). At T1 the participants were asked “Have you changed educational institution the last year?” (yes; no; I'm no longer a student).

#### Personality

At T1 the five factor model's personality traits (i.e., extroversion, agreeableness, conscientiousness, neuroticism, and intellect/imagination) were assessed by Mini-International Personality Item Pool (Mini-IPIP) (McCrae and John, 1992; Donnellan et al., 2006). The Mini-IPIP consists of 20 items (i.e., Four items for each trait), where the respondents are asked to rate the degree specific statements regarding behavior are typical for them. Response alternatives range from “very wrong” (1) to “very right” (5), some items are reversed. Total scores range between 5 and 20 for each trait, where higher scores indicate higher levels of the personality trait in question. The internal reliability of the measurements in the current study was acceptable. The items measuring extroversion, agreeableness, conscientiousness, neuroticism and openness had Cronbach's alphas of 0.83, 0.77, 0.69, 0.75, and 0.74, respectively. Self-monitoring (i.e., attentiveness and adaptability to situational norms) was measured by the revised Self-Monitoring Scale, comprising 13 statements (e.g., “In social situations, I have the ability to alter my behavior if I feel that something else is called for”) in which respondents are asked to rate the applicability of the statements to own behavior (Snyder, 1974; Lennox and Wolfe, 1984). Response alternatives range from “certainly always false” (0) to “certainly always true” (5), some items are reversed. Composite scores range between 0 and 65, where higher scores indicate higher levels of self-monitoring. The internal reliability of the revised Self-Monitoring Scale was acceptable in the current study, with a Cronbach's alpha of 0.82.

#### Social media use

In the first wave participants were asked several questions regarding social media use; if they had an account on a social media site, which sites they used (closed-ended response alternatives of different sites/apps), number of online-friends, and frequency of logins to social media (Karl et al., 2010). Disclosure and exposure of alcohol-related content on social media were assessed by the following questions: “How often do you post content on social media that”: (a) “Refers to positive consequences of alcohol use (e.g., increased pleasure, social cohesion, relaxation)?,” and (b) “Refers to negative consequences of alcohol use (e.g., hangovers, loss of control, hangover anxiety)?” (never; I've done it before, but not lately; less than once a month; every month; a couple of times a month; every week; a couple of times a week; daily or almost daily). Similar questions were asked regarding the frequency of exposure to alcohol-related content. The participants were asked to think only of alcohol-related content that were visible to more than two persons. For the questions regarding exposure to alcohol-related content, the participants were instructed to think only of posts etc. from online-friends or individuals they follow on social media.

#### Alcohol use and alcohol-related cognitions

Alcohol use was assessed at T1 and T2 by the Alcohol Use Disorders Identification Test (AUDIT), comprising 10 items (Bohn et al., 1995; Babor et al., 2001). The respondents are asked to assess their own alcohol use the past year and indicate how often they consume alcohol, how many alcohol units they drink on a typical drinking occasion, how often they drink more than six alcohol units, and how often they experience different adverse consequences related to their alcohol use (e.g., problems controlling consumption, feelings of guilt). The response alternatives vary somewhat, but answers to the different items are given a value between 0 and 4. Total scores on AUDIT range from 0 to 40, where higher scores indicate higher alcohol consumption and more frequent occurrence of alcohol-related harm. Scores over seven are considered to indicate risky drinking (Bohn et al., 1995; Babor et al., 2001). The Cronbach's alpha for AUDIT at T1 and T2 was 0.78 and 0.79, respectively. The measurement of alcohol use at T1 had a strong correlation to the measurements at T2, with a Pearson correlation of 0.80. Descriptive norms for alcohol use and the participants' prototypic apprehension of the typical heavy drinker and the typical sharer of alcohol-related content on social media were assessed at T1. Prototypic apprehensions were measured by the following questions: (a) “What is your overall impression of the typical student that drinks six alcohol units or more on a regular drinking night?,” and (b) “What is your overall impression of the typical student posting alcohol-related content on social media?” Response alternatives ranged from one (extremely negative) to 10 (extremely positive) (Todd and Mullan, 2011). Descriptive norms were assessed by the questions: “Think about the five students you know best. How many of them do you think drink”: (a) “alcohol a couple of times a week?,” (b) “10 alcohol units or more on a typical drinking occasion?,” and (c) “6 alcohol units or more (on the same occasion) a couple of times a week?” (Response range: 0–5 students) (Bohn et al., 1995; Babor et al., 2001; Tickle et al., 2006; Miller et al., 2014). Similar questions were asked to assess descriptive norms for drinking among online-friends, but for these questions the participants were asked to think about the five individuals of which they see most posts from on social media. The answers to the three questions regarding descriptive norms for alcohol use among co-students and among online-friends were summarized. Total scores on descriptive norms for alcohol use among co-students and online-friends thus ranged between 0 and 15, respectively. In the current study the Cronbach's alpha for descriptive norms for alcohol use among co-students was 0.69 and the Cronbach's alpha for descriptive norms for alcohol use among online-friends was 0.72.

### Analysis

Data analyses were conducted with IBM SPSS Statistics 23, R, and Mplus 7. Missing data were deleted listwise. Descriptive analyses were conducted to identify the sample's central tendencies on the study variables. To check for dropout bias, the current sample (i.e., participated at both T1 and T2) was compared to the participants that only participated at T1 on a range of variables by the use of independent sample *t*-tests and chi-square tests. Cohen's *d* or phi coefficients were calculated as an indicator of effect-sizes. By conventional standards Cohen's *d*s of 0.20, 0.50, and 0.80 represent small, moderate, and large effect sizes, respectively (Cohen, 1988). For phi coefficients, 0.10, 0.30, and 0.50 represent small, moderate, and large effect sizes, respectively (Cohen, 1988). Significance tests of difference were conducted for both the independent sample *t*-tests and the chi-square comparisons. Further, we conducted equivalence tests for the independent sample *t*-tests and chi-square tests. Equivalence tests of group difference are based on the assumption that Cohen's *d*s or correlations which are significantly smaller than a given value indicate the absence of any practical meaningful differences (Lakens, 2017). In the current study effect size cut-offs were based on power analyses with 90% power.

Alcohol use (i.e., baseline and later) among those participants who reported to frequently disclose or frequently were exposed to alcohol-related content referring to positive and negative aspects of alcohol were compared to the alcohol use of those who reported low-frequent disclosure or exposure. Baseline alcohol use refers to the participants AUDIT-scores at T1, and later alcohol use refers to AUDIT-scores at T2. Frequent disclosure/exposure was defined as reporting disclosure/exposure monthly or more often.

Crude, partly adjusted, and fully adjusted linear regressions were conducted to investigate the association between disclosure and exposure of alcohol-related content and later alcohol use and changes in alcohol use. Covariates were controlled for (one block at a time) to investigate which factors could explain the relationship between disclosure and exposure and later alcohol use. The dependent variable was AUDIT-score at T2 and change in AUDIT-score (AUDIT T2 minus AUDIT T1). The four main independent variables of interest in the current study were: (i) frequency of disclosure of alcohol-related content depicting positive aspects of alcohol, (ii) frequency of disclosure of alcohol-related content depicting negative aspects of alcohol use, (iii) frequency of exposure to alcohol-related content depicting positive aspects of alcohol, and (iv) frequency to exposure of alcohol-related content depicting negative aspects of alcohol use. The disclosure/exposure variables were dichotomized into frequent (i.e., monthly or more often) disclosure/exposure vs. low-frequent disclosure/exposure. The first regression-models were crude, where no covariates were controlled for. The second models were adjusted for demographics and personality factors (i.e., age, sex, place of birth, religious identification, changes in relationship, and parental status, changes in student status, extroversion, agreeableness, conscientiousness, neuroticism, intellect/imagination, and self-monitoring). The third models were adjusted for other aspects of social media use (i.e., frequency of logins to social media, number of online-friends, having a Snapchat account, and disclosure/exposure of alcohol-related content reflecting positive or negative aspects of alcohol). The fourth models were adjusted for T1 alcohol use (AUDIT-score). The fifth models were adjusted alcohol-related cognitions (i.e., prototypic apprehension of the typical heavy drinker and of the typical sharer of alcohol-related content, descriptive norms for alcohol use among co-students and online-friends). Finally, fully adjusted regressions models were run. All the independent variables, with the exception of changes in relationship-, childcare-, or student status between the first and the second wave, were based on measurements from the first wave. Completely standardized betas are reported for the different regression models as an indicator of effect size; completely standardized betas of 0.10, 0.30, and 0.50 represent small, moderate, and large effect sizes, respectively (Cohen, 1988).

## Results

Table 1 illustrates the sample's characteristics, and a dropout analysis comparing the sample to the participants that only partook at T1. The sample's mean age at T2 was 25.8 years, 64.8% were women, 92.7% were born in Norway, and 83.7% were still students at T2. There were only few significant differences between the sample in the present study (those that participated both at T1 and T2), and those that only participated at T1. Equivalence could not be established for all variables which were included in the dropout analysis. The effect sizes regarding the differences between the group that participated at both T1 and T2 and the group that only participated at T1 were, however, within the range of what is considered as very small. The sample had a mean reduction in AUDIT-score of 0.6 from T1 to T2.

**Table 1:** Sample characteristics and dropout analysis.

	**Participated only at T1 *N* = 6,019**	**Participated at T1 and T2, *N* = 5,217**	**Significance test and effect size of differences**
	**Mean (*SD*) / % (95% CI)**	**Mean (*SD*) / % (95% CI)**	
**DEMOGRAPHICS**			
Age at T2 (i.e., age at T1 + 1 year)	26.0 (6.6)	25.8 (6.3)	Cohen's *d* = 0.025^*E, N.S.*, 2^
Women	62.1% (60.8–63.3%)	64.8% (63.5–66.1%)	Phi = 0.028^*N.E*,^^**^
Born in Norway	92.2% (91.5–92.9%)	92.7% (92.0–93.4%)	Phi = 0.010^*E.*, *N.S.*^
Single at T1	47.6% (46.3–48.9%)	46.9% (45.6–48.2%)	Phi = 0.007*^*E*.^*, ^*N.S.*^
Parent at T1	11.9% (11.0–12.7%)	11.1% (10.2–11.9%)	Phi = 0.012^*E.*, *N.S.*^
Religious at T1	36.0% (34.7–37.2%)	33.4% (32.2–34.7%)	Phi = 0.026^*N.E*,^^**^
Student at T2	–	83.7% (82.7–84.8%)	–
**PERSONALITY**^a^			
Extroversion	14.1 (3.6)	14.0 (3.7)	Cohen's *d* = 0.029^*N.E.*, *N.S.*, 1^
Agreeableness	16.8 (2.8)	16.9 (2.8)	Cohen's *d* = 0.055^*N.E.*,^^**^
Conscientiousness	14.6 (3.2)	14.7 (3.2)	Cohen's *d* = 0.030^*N.E.*, *N.S.*, 1^
Neuroticism	11.1 (3.6)	11.0 (3.7)	Cohen's *d* = 0.021^*E.*, *N.S.*, 2^
Intellect/imagination	14.6 (3.2)	14.6 (3.2)	Cohen's *d* = 0.001^*E.*, *N.S.*, 1^
**SELF-MONITORING**^b^			
Self-monitoring score	40.0 (7.7)	40.1 (7.9)	Cohen's *d* = 0.012^*E.*, *N.S.*, 2^
**ALCOHOL USE**^c^			
AUDIT-score T1	8.2 (4.9)	8.2 (4.9)	Cohen's *d* = 0.013^*E.*, *N.S.*, 1^
AUDIT-score T2	–	7.5 (4.7)	–
Change in AUDIT-score (T2-T1)	–	– 0.6 (3.0)	–
Risky drinking (8 ≤ AUDIT, T1)	53.0% (51.7–54.4%)	53.0% (51.6–54.4%)	Phi = 0.000^*E.*, *N.S.*^
Risky drinking (8 ≤ AUDIT, T2)	–	47.0% (45.6–48.4%)	–
**ALCOHOL-RELATED COGNITIONS**			
Prototypic apprehension of the typical heavy drinker (T1)^d^	5.0 (1.4)	5.0 (1.4)	Cohen's *d* = 0.030^*N.E.*, *N.S.*, 1^
Number of 5 closest co-students that drinks a couple of times a week (T1)	1.9 (1.5)	1.9 (1.5)	Cohen's *d* = 0.004^*E.*, *N.S.*, 1^
Number of 5 closest co-students that typically drink 10 alcohol units or more (T1)	0.8 (1.2)	0.7 (1.2)	Cohen's *d* = 0.063^*N.E.*,^^**^^, 2^
Number of 5 closest co-students that drink 6 units or more a couple of times a week (T1)	1.0 (1.3)	0.9 (1.3)	Cohen's *d* = 0.044 ^*N.E.*,^ ^*^^, 2^
Prototypic apprehension of the typical sharer of alcohol-related content on social media (T1)^d^	4.7 (1.4)	4.7 (1.4)	Cohen's *d* = .016^*E.*, *N.S.*, 1^
Number of 5 closest online-friends that drinks a couple of times a week (T1)	1.8 (1.4)	1.8 (1.5)	Cohen's *d* = .012^*E.*, *N.S.*, 1^
Number of 5 closest online-friends that typically drink 10 alcohol units or more (T1)	0.94 (1.3)	0.86 (1.2)	Cohen's *d* = 0.064^*N.E*,^ ^**^^, 2^
Number of 5 closest online-friends that drink 6 units or more a couple of times a week (T1)	1.1 (1.3)	1.0 (1.3)	Cohen's *d* = 0.058^*N.E*,^ ^**^^, 2^
**SOCIAL MEDIA USE AND ALCOHOL-RELATED DISCLOSURE AND EXPOSURE**			
Frequency of login to social media^e^	6.6 (1.0)	6.7 (0.9)	Cohen's *d* = 0.042^*N.E.*,^ ^*^^, 2^
Number of online-friends	460.0 (272.6)	454.0 (264.3)	Cohen's *d* = 0.023^*E.*, *N.S.*, 2^
Have a Snapchat-account	87.6% (86.7–88.5%)	88.7% (87.8–89.6%)	Phi = 0.018^*N.E.*, *N.S.*^
Frequent disclosure of content reflecting positive aspects of alcohol (T1)^f^	9.4% (8.5–10.2%)	9.6% (8.7–10.4%)	Phi = 0.004^*E*., *N.S*.^
Frequent disclosure of content reflecting negative aspects of alcohol (T1)^f^	2.4% (2.0–2.9%)	2.8% (2.3–3.2%)	Phi = 0.010^*E.*, *N.S.*^
Frequent exposure to content reflecting positive aspects of alcohol (T1)^f^	77.1% (75.9–78.3%)	78.8% (77.6–80.0%)	Phi = 0.021^*N.E.*,^ ^*^
Frequent exposure to content reflecting negative aspects of alcohol (T1)^f^	38.1% (36.7–39.4%)	39.4% (38.0–40.8%)	Phi = 0.014^*E.*, *N.S.*^

*SD Standard deviation, CI Confidence interval, T1 The time of the first wave, T2 The time of the second wave, AUDIT Alcohol Use Disorder Identification Test, ^1^Equal variance assumed, ^2^Equal variance not assumed, E. Equivalent, N.E. Not equivalent, N.S. Not significant*,

* *p < 0.05*,

** *p < 0.01*,

*** *p < 0.001*.

a *Total scores range 5–20 for each trait*.

b *Total scores range 0–65*.

c *Total scores range 0–40*.

.d *Response alternatives = 0 (extremely negative)−10 (extremely positive)*.

e *1 = Seldom/never, 2 = Less than once a week, 3 = 1 time a week, 4 = 2–3 times a week, 5 = 4–6 times a week, 6 = 1–2 times a day, 7 = Over 3 times a day*.

f *Frequent = Monthly or more often*.

The frequent disclosure and exposure groups' alcohol use compared to the low frequent disclosure and exposure groups' use are shown in Tables 2, 3. The frequent disclosure/exposure groups had significantly higher AUDIT-scores at T1 (*p* < 0.001) and T2 (*p* < 0.001) compared to the respective low-frequent disclosure/exposure groups. The frequent disclosure and exposure groups had a higher distribution of risky drinking (AUDIT ≥ 8) at T1 (*p* < 0.001) and T2 (*p* < 0.001), compared to the low-frequent disclosure and exposure groups. Disclosing alcohol-related content at T1 was particularly indicative of risky drinking both at T1 and T2.

**Table 2:** Mean AUDIT-scores among the frequent disclosure and exposure groups.

	**Mean AUDIT-score T1 (*SD*)**	**Mean AUDIT-score T2 (*SD*)**	**Mean change in AUDIT-score from T1 to T2 (*SD*)**
Frequent posting of content reflecting positive aspects of alcohol (T1)	12.50 (4.39)	11.52 (4.76)	−0.87 (3.94)
No frequent posting of content reflecting positive aspects of alcohol (T1)	7.76 (4.63)	7.13 (4.49)	−0.62 (2.91)
Effect size of difference between the two groups	Cohen's *d* = 0.61^***^^a^	Cohen's *d* = 0.57^***^^a^	Cohen's *d* = 0.11^2^
Frequent posting of content reflecting negative aspects of alcohol use (T1)	13.86 (4.56)	12.19 (5.01)	−1.53 (4.03)
No frequent posting of content reflecting negative aspects of alcohol (T1)	8.05 (4.72)	7.42 (4.62)	−0.62 (2.99)
Effect size of difference between the two groups	Cohen's *d* = 0.40^***^^a^	Cohen's *d* = 0.33^***^^a^	Cohen's *d* = 0.44^*^^b^
Frequent exposure to content reflecting positive aspects of alcohol use (T1)	8.74 (4.78)	8.00 (4.68)	−0.70 (3.06)
No frequent exposure to content reflecting positive aspects of alcohol (T1)	6.24 (4.44)	5.84 (4.41)	−0.43 (2.88)
Effect size of difference between the two groups	Cohen's *d* = 0.43^***^^a^	Cohen's *d* = 0.38^***^^a^	Cohen's *d* = 0.13^*^^b^
Frequent exposure to content reflecting negative aspects of alcohol (T1)	9.27 (4.98)	8.46 (4.85)	−0.80 (3.18)
No frequent exposure to content reflecting negative aspects of alcohol (T1)	7.52 (4.58)	6.95 (4.51)	−0.54 (2.91)
Effect size of difference between the two groups	Cohen's *d* = 0.40^***^^b^	Cohen's *d* = 0.35^***^^b^	Cohen's *d* = 0.10^**^^b^

*SD Standard deviation, AUDIT Alcohol Use Disorder Identification Test, T1 The time of the first wave, T2 The time of the second wave, CI Confidence interval, Frequent, Monthly or more often*.

a *Equal variance assumed*.

b *Equal variance not assumed*,

* *p < 0.05*,

** *p < 0.01*,

*** *p < 0.001*.

**Table 3:** Distribution of risky drinking among the frequent disclosure and exposure groups.

	**Risky drinking (8 ≤ AUDIT T1)**	**Risky drinking (8 ≤ AUDIT T2)**
	**(95% CI)**	**(95% CI)**
Frequent posting of content reflecting positive aspects of alcohol (T1)	89.1% (87.1–91.1%)	81.8% (78.1–85.4%)
No frequent posting of content reflecting positive aspects of alcohol (T1)	49.8% (48.8–50.9%)	44.0% (42.5–45.5%)
Effect size of difference between the two groups	Phi = 0.231^***^	Phi = 0.222^***^
Frequent posting of content reflecting negative aspects of alcohol use (T1)	93.1% (89.9–96.3%)	84.7% (78.2–91.3%)
No frequent posting of content reflecting negative aspects of alcohol (T1)	52.5% (51.5–53.5%)	46.6% (45.1–48.2%)
Effect size of difference between the two groups	Phi = 0.129^***^	Phi = 0.123^***^
Frequent exposure to content reflecting positive aspects of alcohol use (T1)	58.8% (57.6–59.9%)	51.7% (50.1–53.4%)
No frequent exposure to content reflecting positive aspects of alcohol (T1)	34.8% (32.8–36.9%)	32.2% (29.2–35.2%)
Effect size of difference between the two groups	Phi = 0.199^***^	Phi = 0.160^***^
Frequent exposure to content reflecting negative aspects of alcohol (T1)	63.1% (61.5–64.7%)	55.9% (53.6–58.3%)
No frequent exposure to content reflecting negative aspects of alcohol (T1)	47.4% (46.1–48.7%)	42.2% (40.3–44.0%)
Effect size of difference between the two groups	Phi = 0.154^***^	Phi = 0.135^***^

*AUDIT Alcohol Use Disorder Identification Test, T1 The time of the first wave, T2 The time of the second wave, CI Confidence interval, Frequent, Monthly or more often*,

*** *p < 0.001*.

### Disclosure and exposure and alcohol use when controlling for covariates

The relationship between frequent disclosure and exposure and later alcohol use, when controlling for different covariates, are shown in Table 4. Controlling for demographic and personality factors reduced the strength of the relationship between frequent disclosure and exposure and later alcohol use, but the reduction was small compared to the reduction seen when the other covariates were controlled for. The association between frequent disclosure and exposure was further reduced when other aspects of social media use was controlled for. Controlling for baseline alcohol use involved the largest weakening of the association between all types of disclosure and exposure and later alcohol use. The association between frequent disclosure and exposure of content referring to positive aspects of alcohol and later alcohol use were, however, still significant even when baseline alcohol use was controlled for. Controlling for alcohol-related cognitions resulted in a weakening of the association between frequent disclosure and exposure and later alcohol use as well. Only the association between frequent disclosure of content referring to positive aspects of alcohol and later alcohol use remained significant when all covariates were controlled for.

**Table 4:** Disclosure/exposure and later alcohol use, while controlling for different covariates (n = 4.342).

**Dependent variables**	**AUDIT-score T2**		**Change in AUDIT-scores (T2–T1)**	
	**B (S.E.)**	**Effect size (Completely standardized Betas)**	**B (S.E.)**	**Effect size (Completely standardized Betas)**
**MODEL 1 (CRUDE)**				
Frequent disclosure of content reflecting positive aspects of alcohol (T1)	4.41 (0.24)^***^	0.28 (0.02)^***^	−0.24 (0.20)	−0.02 (0.02)
Frequent disclosure of content reflecting negative aspects of alcohol (T1)	4.79 (0.47)^***^	0.17 (0.02)^***^	−0.90 (0.37)^*^	−0.05 (0.20)^*^
Frequent exposure to content reflecting positive aspects of alcohol (T1)	2.17 (0.17)^***^	0.19 (0.01)^***^	−0.25 (0.11)^*^	−0.03 (0.01)^*^
Frequent exposure to content reflecting negative aspects of alcohol (T1)	1.52 (0.15)^***^	0.16 (0.02)^***^	−0.24 (0.10)^*^	−0.04 (0.02)^*^
**MODEL 2 (ADJUSTED FOR DEMOGRAPHICS AND PERSONALITY FACTORS**^a^**)**				
Frequent disclosure of content reflecting positive aspects of alcohol (T1)	3.30 (0.24)^***^	0.21 (0.02)^***^	−0.20 (0.20)	−0.02 (0.02)
Frequent disclosure of content reflecting negative aspects of alcohol (T1)	3.17 (0.44)^***^	0.11 (0.02)^***^	−0.82 (0.37)^*^	−0.04 (0.02)^*^
Frequent exposure to content reflecting positive aspects of alcohol (T1)	1.57 (0.15)^***^	0.14 (0.01)^***^	−0.20 (0.11)	−0.03 (0.01)
Frequent exposure to content reflecting negative aspects of alcohol (T1)	0.91 (0.13)^***^	0.09 (0.01)^***^	−0.22 (0.09)^*^	−0.04 (0.02)^*^
**MODEL 3 (ADJUSTED FOR PREVIOUS SOCIAL MEDIA USE AND DISCLOSURE/EXPOSURE**^b^**)**				
Frequent disclosure of content reflecting positive aspects of alcohol (T1)	3.21 (0.26)^***^	0.20 (0.02)^***^	0.02 (0.21)	0.00 (0.02)
Frequent disclosure of content reflecting negative aspects of alcohol (T1)	1.83 (0.51)^***^	0.06 (0.02)^***^	−0.80 (0.40)^*^	−0.04 (0.02)^*^
Frequent exposure to content reflecting positive aspects of alcohol (T1)	1.09 (0.17)^***^	0.10 (0.01)^***^	−0.15 (0.11)	−0.02 (0.02)
Frequent exposure to content reflecting negative aspects of alcohol (T1)	0.57 (0.14)^***^	0.06 (0.02)^***^	−0.15 (0.10)	−0.02 (0.02)
**MODEL 4 (ADJUSTED FOR PREVIOUS ALCOHOL USE**^c^**)**				
Frequent disclosure of content reflecting positive aspects of alcohol (T1)	0.82 (0.19)^***^	0.05 (0.01)^***^	0.83 (0.19)^***^	0.08 (0.02)^***^
Frequent disclosure of content reflecting negative aspects of alcohol (T1)	0.33 (0.36)	0.01 (0.01)	0.33 (0.36)	0.02 (0.02)
Frequent exposure to content reflecting positive aspects of alcohol (T1)	0.29 (0.11)^**^	0.03 (0.01)^**^	0.28 (0.11)^**^	0.04 (0.01)^**^
Frequent exposure to content reflecting negative aspects of alcohol (T1)	0.14 (0.09)	0.02 (0.01)	0.14 (0.09)	0.02 (0.02)
**MODEL 5 (ADJUSTED FOR PREVIOUS ALCOHOL-RELATED COGNITIONS**^d^**)**				
Frequent disclosure of content reflecting positive aspects of alcohol (T1)	2.23 (0.24)^***^	0.14 (0.02)^***^	0.07 (0.21)	0.01 (0.02)
Frequent disclosure of content reflecting negative aspects of alcohol (T1)	2.11 (0.45)^***^	0.07 (0.02)^***^	−0.57 (0.38)	−0.03 (0.02)
Frequent exposure to content reflecting positive aspects of alcohol (T1)	1.05 (0.15)^***^	0.09 (0.01)^***^	−0.11 (0.11)	−0.02 (0.02)
Frequent exposure to content reflecting negative aspects of alcohol (T1)	0.64 (0.13)^***^	0.07 (0.01)^***^	−0.13 (0.10)	−0.02 (0.02)
**MODEL 6 (FULLY ADJUSTED)**				
Frequent disclosure of content reflecting positive aspects of alcohol (T1)	0.63 (0.20)^**^	0.04 (0.01)^**^	0.64 (0.20)^**^	0.06 (0.02)^**^
Frequent disclosure of content reflecting negative aspects of alcohol (T1)	−0.25 (0.37)	−0.01 (0.01)	−0.25 (0.37)	−0.01 (0.02)
Frequent exposure to content reflecting positive aspects of alcohol (T1)	0.15 (0.10)	0.01 (0.01)	0.14 (0.10)	0.02 (0.01)
Frequent exposure to content reflecting negative aspects of alcohol (T1)	−0.04 (0.09)	−0.00 (0.01)	−0.04 (0.09)	−0.01 (0.02)

*Covariates are adjusted for one by one. Fully adjusted analyses include all covariates. AUDIT Alcohol Use Disorder Identification Test, S.E. Standard Error, Frequent = monthly or more often, reference category: less than monthly, OR Odds ratio, CI Confidence interval, T1 The time of the first wave, T2 The time of the second wave*,

* *p < 0.05*,

** *p < 0.01*,

*** *p < 0.001*.

a *Age, sex, place of birth, religious identification (T1), relationship status (T1 and T2), student status (T2), parental status (T1 and T2), extroversion (T1), agreeableness (T1), conscientiousness (T1), neuroticism (T1), intellect/imagination (T1), and self-monitoring (T1)*.

b *Frequency of logins to social media (T1), number of online-friends (T1), having a Snapchat account (T1), and disclosure/exposure of alcohol-related content reflecting positive or negative aspects of alcohol (T1)*.

c *Alcohol use T1 (AUDIT-score)*.

d *Prototypic apprehension of the typical heavy drinker and of the typical sharer of alcohol-related content (T1), descriptive norms for alcohol use among co-students and online-friends (T1)*.

Participants which frequently disclosed content referring to negative aspects of alcohol or were frequently exposed to content referring to positive or negative aspects of alcohol had a significant reduction in their AUDIT-scores from T1 to T2, compared to the respective low-frequent disclosure and exposure groups. This reduction was eliminated when baseline alcohol use was controlled for. Participants who reported frequent disclosure of content referring to positive aspects of alcohol experienced an increase in later alcohol use, compared to the participants that reported low-frequent disclosure of such content.

Regression models with large sample sizes and single items measurements could involve an increased risk of conducting type I errors (Westfall and Yarkoni, 2016). The regression models were subsequently conducted using structural equation models to ensure that the results found in the regression models were robust. Alcohol use at T1, the five factor model's personality traits, self-monitoring, descriptive norms for alcohol use among co-students and among online-friends, and alcohol use at T2 were latent variables in the structural equation models. These models (results not shown) yielded similar results as the reported regression models.

## Discussion

Our findings suggest that frequent disclosure and exposure to alcohol-related content on social media is positively associated with both baseline and later alcohol use. This supports the notion that social media can be a suitable arena for detecting problematic alcohol usage. In particular, disclosure of alcohol-related content was related to high alcohol use, which is in line with previous research (Miller et al., 2014; Westgate et al., 2014). It should be noted that the whole sample demonstrated a reduction in alcohol use over time. The frequent disclosure and exposure-groups had higher alcohol consumption compared to the low-frequent disclosure and exposure groups. Some of the disclosure and exposure-groups did, however, also have a larger reduction in alcohol use (when no covariates were controlled for), compared to the respective low-frequent disclosure and exposure groups. We speculate that the larger reduction in alcohol use observed among some of the disclosure and exposure-groups could be explained by their initial high alcohol use, as individuals tend to regress toward group means over time (Bland and Altman, 1994). In line with this, the disclosure and exposure-groups relative reduction in alcohol use disappeared when baseline alcohol use was controlled for. Frequent disclosure of content referring to positive aspects of alcohol use was associated with stable or slightly increasing alcohol use over time (when all covariates were controlled for), compared to the reduction in alcohol use found among participants with low-frequent disclosure of such content.

The relationship between disclosure and exposure and baseline and later alcohol use differed based on the type of alcohol-related content shared or seen. Disclosure of content referring to negative aspects of alcohol use was a stronger indicator of alcohol use than disclosure of content referring to positive aspects of alcohol use, when no covariates were controlled for. The experience of adverse effects is considered to be an important indicator of problematic alcohol use (Babor et al., 2001). Accordingly, disclosing content related to negative aspects of alcohol may serve as a measure of problematic alcohol usage. However, disclosing content referring to positive aspects of alcohol use were identified as a stronger indicator of later alcohol use when the different covariates were controlled for, which may suggest that disclosure of such content predicts stabile or increasing alcohol use when other factors (e.g., demographics) are hold constant. In addition, exposure to alcohol indicated later alcohol use; here the relationship was strongest for content reflecting positive aspects of alcohol use. However, the relationship between exposure and later alcohol use was not significant when all covariates were controlled for.

### Common factors did not explain the relationship between disclosure and exposure and later alcohol use

The association between disclosure and exposure and later alcohol use was mitigated when controlling for demographics and personality factors, and for other aspects of social media use. Disclosure and exposure have been or may be linked to certain demographic, personality, and social media use factors (e.g., being single, extroversion, strong social media engagement) which again have been linked to increased alcohol use (Merenakk et al., 2003; Andersson et al., 2007; Egan and Moreno, 2011; Beullens and Schepers, 2013; Nedregård and Olsen, 2014; Westgate et al., 2014; Erevik et al., 2017). The current results nevertheless suggest that the relationship between disclosure and exposure and later alcohol use cannot be fully attributed to such confounding factors.

### Disclosure and exposure was strongly associated with baseline alcohol use and alcohol-related cognitions

The association between disclosure and exposure and later alcohol use was substantially reduced when adjusting for alcohol use at baseline. Few studies have controlled for baseline alcohol use, but the current findings (i.e., small or no effect of exposure on later alcohol use) are similar to the findings from comparable studies (Huang et al., 2014a,b; Boyle et al., 2016). Disclosure as an indicator of later alcohol use has not been investigated longitudinal before, while controlling for baseline alcohol use. The current findings suggest that disclosure and exposure primarily reflects baseline alcohol use, that high alcohol use predicts both disclosure and exposure to alcohol as well as further alcohol use. On the other hand, it should be noted that the measurements in the current study were separated by 1 year. It is possible that the temporal relationships between disclosure and exposure and later alcohol use are either too short-lived or take longer time to become established than the one-year follow-up time used in present study. In addition, the strong correlation between alcohol use at T1 and T2 and the clear association between alcohol disclosure/exposure on social media and high alcohol use, suggest that these behaviors might be intertwined and reciprocally reinforcing. Thus, the issue of directionality seems hard to determine with the current design. Individuals with high alcohol use may display and be exposed to more alcohol use in both online and offline settings and continue to have a high alcohol use over time. Hence, the potential separate effects of online alcohol disclosure/exposure on further alcohol use might be hard to detect. The current results nonetheless suggest that alcohol disclosure/exposure's potential effects on further alcohol are likely to be small in populations were alcohol habits are pre-established, e.g., college/university student populations. Adjusting for baseline norm perceptions and attitudes also resulted in a weakening of the association between disclosure and exposure and later alcohol use. The current finding suggest as such that the association between alcohol disclosure/exposure on social media and later alcohol use, could in part be explained by an association between disclosure/exposure and lenient alcohol-related cognitions (Westgate et al., 2014).

### Disclosing positive alcohol content may influence later alcohol use

Frequent disclosure of content reflecting positive aspects of alcohol use was the only independent variable that predicted later alcohol use, when all covariates were controlled for. Disclosure of content related to positive aspects of alcohol use may reflect attitudes or experiences regarding alcohol use not measured in the current study. Disclosure of positive alcohol-related content could suggest that the individuals have positive attitudes toward alcohol or alcohol-related cognitions, which were not detected by the alcohol-related cognitions questions included in the present study (D'Angelo et al., 2014; Westgate et al., 2014). Disclosures of alcohol-related content referring to positive aspects of alcohol could also indicate that the individual is experiencing pleasurable effects of alcohol. Positive alcohol-related cognitions and the experience of positive consequences are further considered as important motivational determinants of further alcohol use (Park, 2004; Zimmermann and Sieverding, 2010; Lee et al., 2011).

Disclosing content related to positive aspects of alcohol might, however, also represent a causal influence on later alcohol use. Posting alcohol-related content referring to positive aspects of alcohol use may make the senders' attitudes toward alcohol more positive, through mechanisms such as self-identification with own postings, potential self-fulfilling prophecies, and cognitive dissonance (Merton, 1948; Festinger, 1962; Bem, 1972; D'Angelo et al., 2014). Disclosures of positive alcohol-related content may also cause a later increase in alcohol use through likes and other types of virtual appraisals acting as positive reinforces (Skinner, 1953; D'Angelo et al., 2014; Groth et al., 2017). A previous study found disclosure of content referring to positive aspects of alcohol to yield more likes than disclosure of content referring to negative aspects of alcohol (Beullens and Schepers, 2013). The increased number of likes associated with alcohol-related content reflecting positive aspects of alcohol may explain why disclosure of this type of content predicted later alcohol use, even when all covariates were adjusted for.

### Limitations and strengths

The present study is not without limitations. The measurements are based on self-report, and responses to self-report questions may be affected by social desirability and recall biases (Raphael, 1987; Gnambs and Kaspar, 2015). Another limitation with the current study is that some concepts were measured by single-items (e.g., prototypic evaluations, specific type of alcohol disclosure/exposure), which could make the results more likely to be affected by measurements errors (Nunnally, 1978). In addition, some variables that may explain the relationship between disclosure/exposure and alcohol use (e.g., parents' alcohol use) were not included in the study. Finally, the study's sample consisted of Norwegian students, which may limit the generalizability of the current findings. A major strength with the present study is the longitudinal design, and the study can hence give an indication of directionality and causality (Rutter, 1988). The high correlation between alcohol use at T1 and T2, and the strong association between concurrent alcohol use and alcohol disclosure/exposure do, however, hamper conclusions regarding directionality. Further, it is important to note that causality cannot be established (only indicated) by the current research design (Cohen et al., 2013). The study provides new knowledge regarding the relationship between different types of alcohol-related content and later alcohol use, and the importance of different covariates in this relationship. The comprehensive set of covariates included and controlled for, and the large sample size are further strengths of the present study, which clearly distinguishes the current study from any previous studies in the research field.

## Conclusions

Frequent disclosure and/or exposure to alcohol-related content indicate consecutive high alcohol intake. The association between disclosure/exposure and subsequent alcohol use was considerably weakened when baseline alcohol use was adjusted for. This finding might suggest that disclosure/exposure primarily reflects baseline alcohol use. Alcohol disclosure/exposure on social media and high alcohol use might, however, be intertwined and reciprocally reinforcing behaviors making the separate effects of online alcohol disclosure/exposure on further alcohol use hard to detect. More research and experimental studies are required to make final conclusions regarding directionality and causality in the relationship between alcohol disclosure/exposure on social media and alcohol use. Disclosing content reflecting positive aspects of alcohol may have a self-enhancing effect on the sharers' alcohol use and can predict stable or slightly increasing alcohol consumption over time when other factors (e.g., baseline alcohol use) are hold constant. The relationship between disclosure of content referring to positive aspects of alcohol and later alcohol use may, however, also be explained by potential third variables not included in the current study, e.g., positive alcohol experiences.

## Author contributions

Conceived and designed the research: SP, EE, TT, and CA. Preformed the research: EE, SP, and TT. Analyzed the data: EE, TT, ØV, and SP. Contributed to the writing of the manuscript: EE, SP, CA, ØV, and TT.

### Conflict of interest statement

The authors declare that the research was conducted in the absence of any commercial or financial relationships that could be construed as a potential conflict of interest.
